# Measuring results of humanitarian action: adapting public health indicators to different contexts

**DOI:** 10.1186/s13031-022-00487-5

**Published:** 2022-10-14

**Authors:** Chiara Altare, William Weiss, Marwa Ramadan, Hannah Tappis, Paul B. Spiegel

**Affiliations:** 1 Center for Humanitarian Health, Johns Hopkins Bloomberg School of Public Health, Baltimore, MD USA; 2 Jhpiego, Baltimore, MD USA

**Keywords:** Measurement methods, Humanitarian settings, Performance, Monitoring and evaluation, Public health indicators

## Abstract

Humanitarian crises represent a significant public health risk factor for affected populations exacerbating mortality, morbidity, disabilities, and reducing access to and quality of health care. Reliable and timely information on the health status of and services provided to crisis-affected populations is crucial to establish public health priorities, mobilize funds, and monitor the performance of humanitarian action. Numerous efforts have contributed to standardizing and presenting timely public health information in humanitarian settings over the last two decades. While the prominence of process and output (rather than outcome and impact) indicators in monitoring frameworks leads to adequate information on resources and activities, health outcomes are rarely measured due to the challenges of measuring them using gold-standard methods that are difficult to implement in humanitarian settings.

We argue that challenges in collecting the gold-standard performance measures should not be a rationale for neglecting outcome measures for critical health and nutrition programs in humanitarian emergencies. Alternative indicators or measurement methods that are robust, practical, and feasible in varying contexts should be used in the interim while acknowledging limitations or interpretation constraints. In this paper, we draw from existing literature, expert judgment, and operational experience to propose an approach to adapt public health indicators for measuring performance of the humanitarian response across varied contexts.

Contexts were defined in terms of parameters that capture two of the main constraints affecting the capacity to obtain performance information in humanitarian settings: (i) access to population or health facilities; and (ii) availability of resources for measurement. Consequently, 2 × 2 tables depict four possible scenarios: (A) a situation with accessible populations and with available resources; (B) a situation with available resources but limited access to affected populations; (C) a situation with accessible populations and limited resources; and (D) a situation with both limited access and limited resources.

Methods and data sources can vary from large population-based surveys, rapid assessments of populations or health facilities, routine health management information systems, or data from sentinel sites in the community or among facilities. Adapting indicators and methods to specific contexts of humanitarian settings increases the potential for measuring the performance of humanitarian programs beyond inputs and outputs by assessing health outcomes, and consequently improving program impact, reducing morbidity and mortality, and improving the quality of lives amongst persons affected by humanitarian emergencies.

**Supplementary Information:**

The online version contains supplementary material available at 10.1186/s13031-022-00487-5.

## Background

Humanitarian crises represent a significant public health risk factor for affected populations by reducing access to quality health services and consequently affecting mortality, morbidity and quality of life. Reliable and timely information on the health status and services provided to crisis-affected populations is crucial to establish public health priorities, mobilize funds, and monitor performance of humanitarian action [1]. However, governments and humanitarian organizations are rarely able to measure how well humanitarian action is achieving the expected results, particularly in non-camp settings [2]. Measuring the effectiveness of humanitarian assistance is fraught with challenges including availability of resources (e.g., funding for monitoring activities, system readiness and human capacity), and access to affected populations or health facilities. Furthermore, the prominence of process and output (rather than outcome and impact) indicators in monitoring frameworks may lead to adequate information on resources distributed and activities implemented, but little information about the effectiveness of humanitarian action [3]. Performance measures are instrumental to ensure humanitarian action meets population needs, utilizes limited resources effectively and ensures accountability to affected populations.

Numerous inter- and intra-agency initiatives have contributed to improving public health information in emergency settings by providing guidance in terms of indicators (e.g., Global Health Cluster Core Indicators [4], the Sphere project) [5], methods to measure them (e.g., survey methodologies such as the Standardized Monitoring and Assessment of Relief and Transitions (SMART) methodology [6], the United Nations High Commissioner for Refugees’ (UNHCR) Standardized Expanded Nutrition Survey (SENS) [7] and Health Access and Utilization Survey (HAUS) [8], and standardized tools and mechanisms to collect, capture and report on them (e.g., Global Health Cluster work on Public Health Information Services [9], UNHCR’s Integrated Refugee Health Information System – iRHIS [10], and KoBo toolbox [11]). These global efforts have led to increased availability and standardization of information in various areas such as nutrition, mortality, and epidemics [12].

Despite these efforts, outcome and impact indicators are still not consistently measured and reported, nor used for evidence-based decision-making in most conflict and forced displacement settings because they are difficult to measure by a ‘one-size-fits-all’ “gold” standard methodology. In our work we refer to a “gold standard” as the most used or most rigorous indicator and/or method to measure a certain outcome, often in stable development settings. These include, for example, population based surveys assessing the prevalence of a certain disease, or observation of a certain practice in case management or of a certain behavior. We argue that challenges in collecting the gold standard performance measure should not be a rationale for neglecting outcome measures for critical health and nutrition programs in humanitarian emergencies. Rather, strategies for adapting gold standard indicators and/or measurement methods should be established so that, in the interim, alternative performance measures are available and robust, practical, and feasible in varying contexts. In this paper, we draw from existing literature, expert judgment, and operational experience to present an approach for adapting a preliminary reference set of performance indicators to various contexts. In addition, we reflect on limitations and interpretation constraints of the associated measurement methods that need to be adapted for humanitarian contexts with varying levels of population access and human or financial resources. This debate article is composed of five parts: first, we review current practice in measuring public health outcome indicators in humanitarian settings; second, we attempt to define alternative indicators for different humanitarian scenarios based on access to population and resource availability and we provide preliminary reference indicators for a variety of public health domains; third, we propose consolidated methods across indicators according to the same humanitarian scenarios; we then discuss methodological implications and limitations; and, fifth, we conclude by summarizing key messages and opening the discussion to the readers.

## Public health indicators in use

We reviewed peer-reviewed articles and grey literature to take stock of the current and previously used public health outcome indicators to assess results of humanitarian action (see supplementary material for details about the literature search strategy). We included both peer-reviewed articles published in English during the last 30 years (1988–2018) as well as operational, monitoring and strategic documents of humanitarian actors; the latter included monitoring frameworks, indicator and measurement guidelines from United Nations (UN) agencies, non-governmental organizations (NGOs), and donors. We also reviewed existing lists of indicators and standards, as well as humanitarian response plans from two crises (South Sudan and Yemen). The scope of the search included maternal and child health, nutrition, communicable and non-communicable diseases, sexual and reproductive health, injuries and disabilities, and mental health. Health-related components of water, sanitation and hygiene (WASH) topics, such as hand washing practice were also included, while infrastructure-related aspects, such as the performance of WASH interventions were excluded. Details about search strategies are outlined in supplementary material . We conducted backward citation searching from identified documents.

We identified 800 unique indicators that were reorganized around *thematic constructs and sub-constructs* (e.g., non-communicable diseases is a construct, and diabetes is a sub-construct) and three *dimensions*: (i) Health status, defined as the population health condition and outcome of a health service; (ii) Quality, narrowly defined as compliance with protocol or, if not possible, system readiness (recognizing that provision of evidence-based care is only one component of quality care, and quality care must also be timely, person-centered, safe, efficient, coordinated and equitable [13]); and (iii) Coverage, defined as the proportion of people who need a service receiving it or the proportion of people practicing a given behavior. A complete list of thematic constructs and sub-constructs is included in supplementary material (table S2). Indicators related to health status and service coverage each represented about 40% of all identified indicators. Quality indicators were 20% of the total. Of the total indicators, 10% were reported by more than one source; 3% of the indicators were found only in peer-reviewed literature and not in operational guidance.

Identified indicators were then consolidated based on the literature, expert judgement and operational experience. We discussed indicators in terms of utilization, perceived validity, feasibility and actionability, both among the authors and with more than 100 humanitarian actors working in a variety of organizations (national government offices, UN and NGOs) in five different crises (North and South Kivu provinces in the Democratic Republic of Congo (DRC); Juba and Wau state in South Sudan; Kampala and Arua settlements in Uganda; Amman and refugee camps in Jordan; and Gaziantep in Turkey). These settings were chosen to represent a variety of crises (longer and shorter-term, outside and inside camps, different population profiles, varying levels of resources). At each site, semi-structured qualitative interviews were conducted with health and nutrition officers of UN agencies, NGOs and national authorities to understand the availability of data elements to calculate indicators, monitoring challenges, data utilization and feasibility of our proposed approach. Interviews were complemented with health facilities visits and review of patient registers.

## Alternative indicators or methods according to various humanitarian contexts

The literature review and interviews showed that the feasibility of measuring the performance of humanitarian programs around a particular health construct (e.g., mental health) depends on the context. Each humanitarian setting is characterized by features (e.g., level of urgency, time and resource availability, human capacity, security constraints, access) that dictate which indicators and which measurement methods are possible. Context, therefore, acts as a mediator that impacts the choice of how a construct and dimension can be measured.

**Fig. 1:**
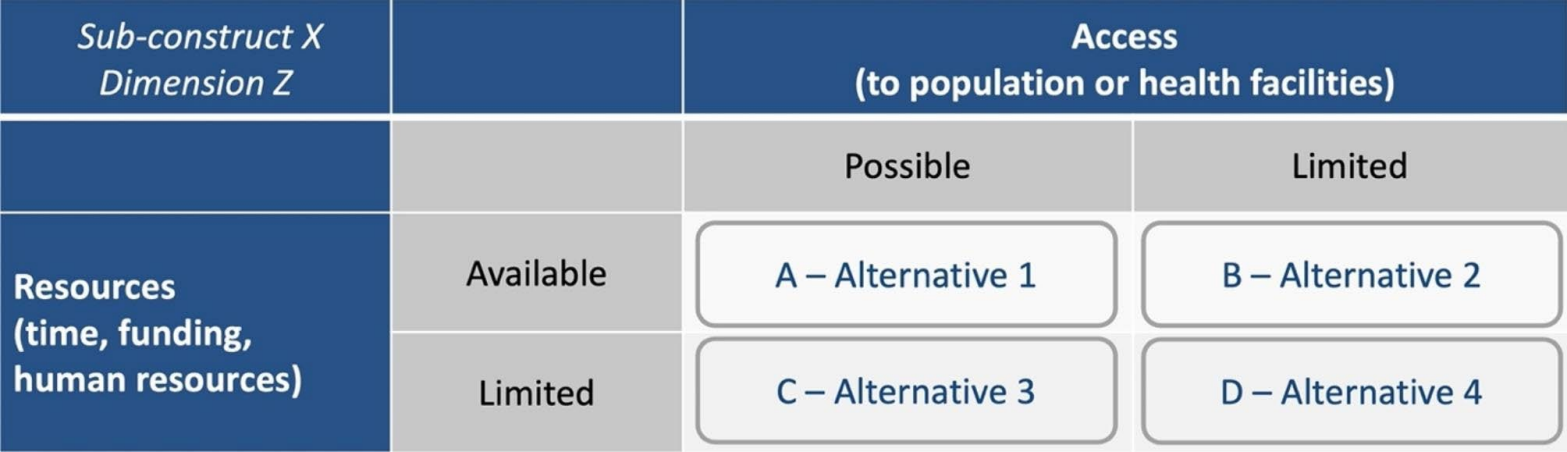
Operationalization of indicators and measurement methods according to settings defined in terms of access to population or health facilities AND level of available resources

Specific to each sub-construct/dimension pair, we attempted to operationalize indicators for various humanitarian contexts by identifying alternative indicators and/or measurement methods that are appropriate for varying scenarios. Context was defined in terms of two parameters that capture two of the main constraints affecting the capacity to obtain data: (i) access to population or health facilities (i.e., the capacity to reach the population that can be hindered by security reasons or remoteness); and (ii) resource availability (including the level of functionality of the health information system, financial and human resources, and time). While we recognize that this is a simplification of reality, working with two main parameters allowed us to utilize a 2 × 2 table to depict four possible scenarios: (A) a situation with access to affected populations and with adequate resources available for performance monitoring; (B) a situation with adequate resources available but with limited physical access; (C) a situation with access to affected populations but limited resources; and (D) a situation with both limited access and limited resources (Fig. 1).

For each sub-construct and dimension of the framework, one reference indicator was identified from the desk review and expert judgment (including both external consultations and internal review), and then adapted to the four scenarios to provide a reference indicator and method that was feasible in each. In so doing, we tailored indicators according to the available resources and access to help ensure that the performance of humanitarian action could be measured, even in settings where the gold standard method was not feasible (e.g., often a population-based data source).

**Fig. 2:**
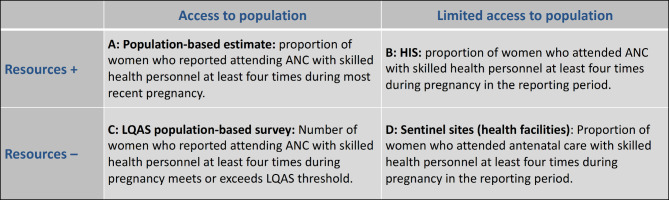
Reference indicators to measure antenatal care coverage

**Fig. 3:**
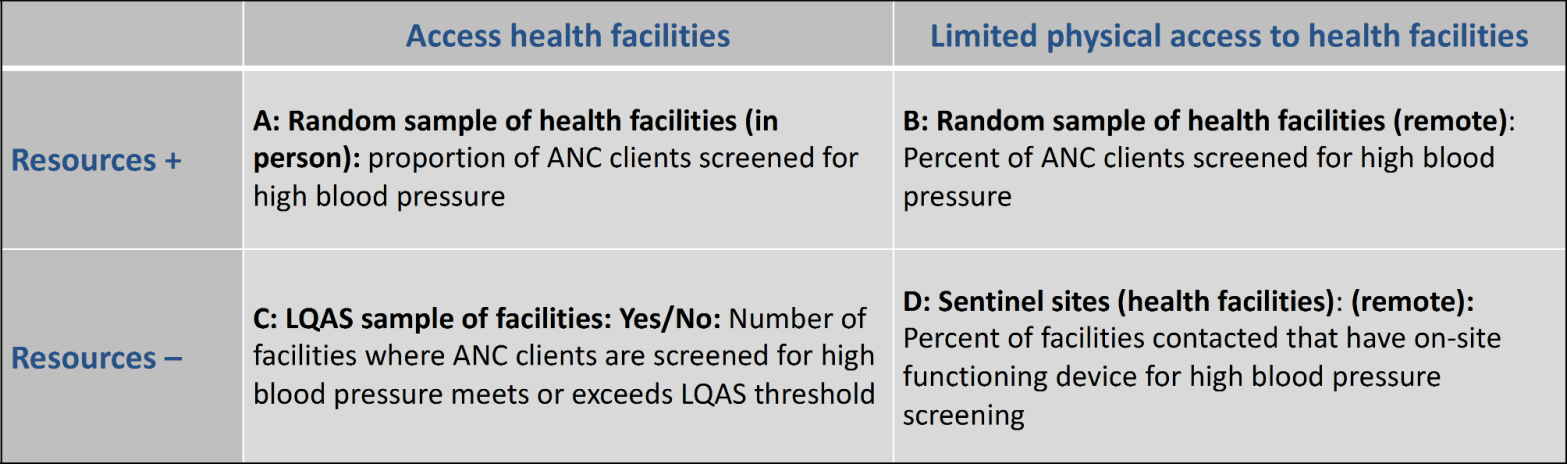
Reference indicators to measure antenatal care quality

We drafted 2 × 2 tables of reference indicators for 56 public health outcomes encompassing morbidity (age and cause specific), nutritional status, acute health care services (maternal, sick infant, newborn and child, gender based violence, trauma and undernutrition); chronic health care services (hypertension, diabetes, HIV, TB, mental health); preventative services (antenatal care, family planning, immunization, labor, safe abortion care, postnatal care); preventive practices (breastfeeding, handwashing, care seeking); and health system building blocks (service delivery, essential medicine, health information system, infection prevention and control). The complete list of reference indicators is available in table S3 in the supplementary material. The goal of this exercise was to provide one indicator for each dimension and sub-construct to guide how indicator definitions and methods can be systematically adapted to monitor key health constructs at the outcome level across varying humanitarian contexts. The provided reference indicators align with international practice/standards as much as possible and were based on the review and expert judgment discussed above. We describe in Figs. 2 and 3 two examples of the 2 × 2 tables showing how coverage and quality of antenatal care (ANC) can be measured in different settings.

For example, coverage (dimension) of ANC (sub-construct of maternal health, a construct) is measured as the proportion of women who reported attending ANC with skilled health personnel at least four times during the most recent pregnancy (Fig. 2). This indicator is proposed to be used in all four scenarios, however, the measurement method differs: population-based estimate when resources and access are available (cell A); routine health information system (HIS) when resources are available but access is limited (cell B); lot quality assurance sampling (LQAS)^1^ where resources are limited but access is possible (cell C); and contacting (perhaps remotely) sentinel health facilities when access and resources are limited (cell D).

Figure 3 presents another example, a 2 × 2 table for quality (another dimension) of ANC. As quality is defined in this example in terms of compliance with clinical protocol, its measurement relies mainly on data from health facilities. We suggest blood pressure (BP) measurement as tracer indicator as high BP is a risk factor for adverse pregnancy outcomes and BP should be monitored at each ANC visit. BP screening is proposed in three of the four scenarios, each with a different reference measurement method depending on the context. These include random sampling of health facilities when access and resources are available (cell A); the use of routine health data or a remote sampling of health facilities when access is limited but resources are available (cell B); and LQAS when access is possible, but resources limited (cell C). Unlike for ANC coverage, the reference indicator for cell D is different as compliance with protocol was deemed too difficult to measure in a context with limited access and resources. It is, therefore, recommended to focus on system readiness instead and use the presence of a functioning device for BP monitoring as an indication of ANC quality. In some cases, it may still be possible to obtain information on the frequency of use of the screening device.

## Operationalization of indicators for various humanitarian contexts

During the creation of the 2 × 2 tables, it became evident that even in challenging contexts, the key constructs/dimensions relevant to understanding the performance of humanitarian assistance programs can be assessed if one was willing to use other methods and data sources as compared to those required for the gold standard indicator that was no longer considered feasible to measure.

**Fig. 4:**
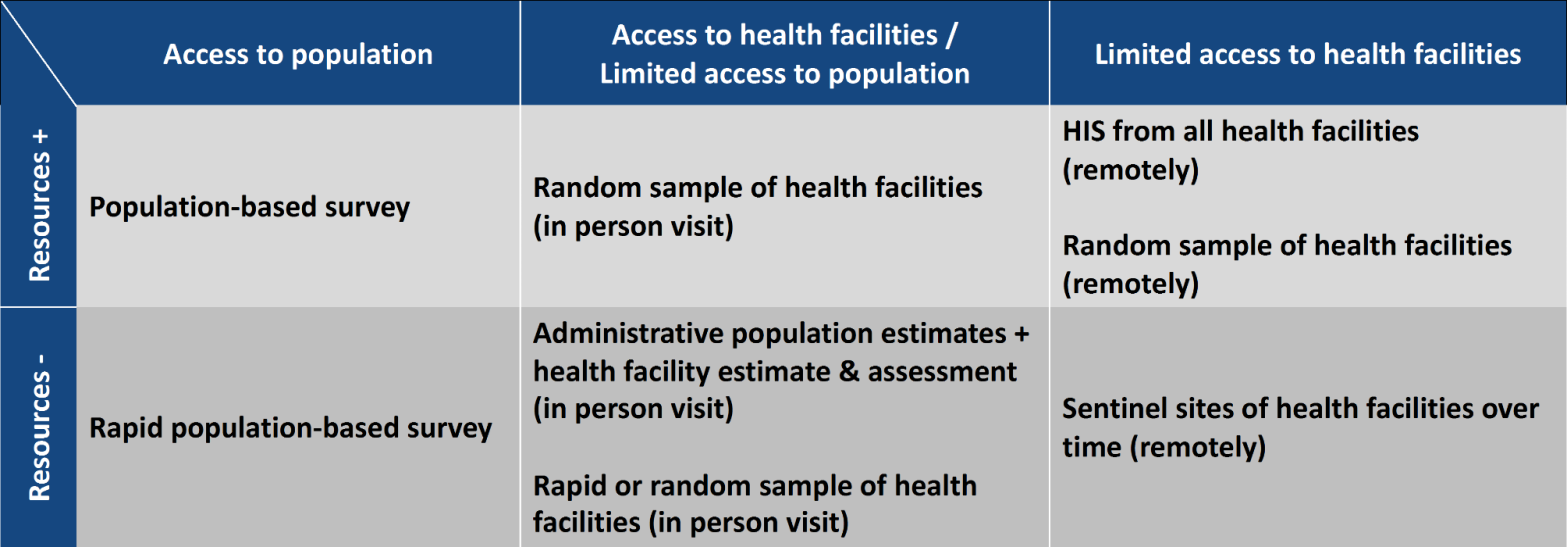
Overview of measurement methods by level of access and resources

Due to the complexity of data collection in challenging and insecure humanitarian settings, we propose a 2 × 2 table framework to pragmatically guide the choice of methods for assessing key constructs central to understanding performance of humanitarian programs in a particular context. Population-based surveys are most often recommended for assessing population health status and service coverage when access to the population is granted and resources are available. An alternative solution when resources are limited is to use LQAS to classify levels of coverage or quality of a relevant construct. When access to the population is not granted, health facilities may represent an important source of information. Sampling approaches can be applied to health facilities and can be combined with population estimates as denominators. When in-person visits are possible, reviewing medical records can be a way to assess quality of care. Finally, when access is limited or completely absent, remote monitoring of all health facilities or with a sentinel site approach can allow to better understand performance of a relevant construct. Figure 4 summarizes methods used in humanitarian settings and provides a framework to guide the selection of the most appropriate method according to context. Methods can be imagined as a continuum of options to choose from according to access and resource levels, recognizing that multiple approaches exist to generate evidence [3].

All the methods that we propose are already being used by various humanitarian actors, yet often in an unsystematic and inconsistent way. Household surveys are a well-known method of measurement, but implementation has varied across countries. In Jordan, such surveys were rarely conducted, although they are considered feasible because access to the population is not a constraint. However, in North-Western Syria, household surveys were regularly undertaken, even during the crises. In South Sudan, a limited number of partners were responsible for conducting nutrition surveys in the country, and their frequency decreased due to limited funding. Finally, surveys in DRC were rarely conducted as they were considered too expensive. In all situations, population-based surveys were reported as highly dependent on external funding.

LQAS is not utilized as commonly in the humanitarian field as the other methods, but it has been used both at a local level by NGOs in Darfur, Sudan [14] and at both local [15] as well as national levels in South Sudan [16] and DRC. Health facility surveys via sampling were not routinely conducted, although often considered feasible. Organizations tended to support only a few health facilities, and knew which services were provided. Therefore, they generally visited all heath facilities they supported. If access was limited, they postponed or canceled the visit, but applying a sampling strategy was uncommon. This is clearly different from ad-hoc, national-level health facility assessments such as the Service Availability and Readiness assessment (SARA) or the Service Provision Assessment (SPA) which rely on a large sample of health facilities.

The existence of updated population figures varies by country and remains a challenge in many humanitarian settings. If a recent census was unavailable, projections or alternative data sources were used (e.g., sampling frames from distributions, or “health counts”). The estimates of displaced populations were usually from UN agencies, especially for refugees. Estimating the numbers of internally displaced persons was more challenging, and was often not integrated into population estimates in a timely manner, reducing the level of reliability of the estimates. Organizations tended to rely on population estimates from UN agencies or national authorities for planning and budget purposes, but rarely for calculating outcome or impact indicators.

## Methodological issues and limitations

Complementary to the extensive existing guidance [9] on the individual methods (e.g., survey and sampling methods [6, 7], use of routine health data [17],and estimation of population denominators [18]),  we provide systematic guidance on how to choose indicators and methods according to availability of human and technical resources as well as access to the affected population.

There are three methodological considerations that merit further discussion. First, there is a trade-off between the feasibility of a measurement method versus the representativeness and comparability of results. Depending on how the results are to be used, one may be more important than the other, and this can change over time. Population-based surveys are often considered the gold standard as they provide a representative snapshot of a situation that can inform needs and programs and are often comparable across settings. However, classification of situations may be sufficient for certain programmatic or strategic decisions, without needing a point estimate and precise confidence interval of a certain measure. LQAS, as a classification approach, could therefore represent a less expensive alternative to standard population-based surveys when the latter are not feasible.

Second, different measurement methods require different interpretation and aggregation approaches. The same indicator estimated with a survey sample cannot be interpreted as one estimated with health facility data, as the populations captured by these two methods may be different. Yet, each estimate may provide useful information especially when observed over time and complemented with contextual information such as disease seasonality, population displacement, and conflict. Direct comparison of estimates generated with different methods should be avoided, but interpretations can be utilized to understand the performance of programs, while clearly recognizing the limitations and biases of each method.

Third, a main challenge of the proposed approach is how to transition between methods when context changes, given that direct comparison of indicators measured with different methods should be avoided. We propose analyzing results and gauging the situation at the sub-construct level, instead of at the indicator level, and integrating qualitative appraisals of the situation to provide complementary information to the quantitative assessment. Measurement of performance is multifaceted and should consider not only multiple outcomes, but also different types of data to provide a comprehensive understanding of the results.

Besides these methodological considerations, this work can contribute to the ongoing efforts to increase attention to the quality of health care in humanitarian settings [13]. We have systematically identified reference indicators for the quality of preventative, acute and chronic health services that can be integrated into routine monitoring in many difficult settings. Expert groups can debate the reference measures for their areas of expertise [19] and adapt or disaggregate them as needed, although the proposed reference indicators are aligned with current global guidance and can be used as starting point in the interim. However, our approach only attempts to capture one of the dimensions of quality of care from the provider’s perspective (provision of evidence-based care), without addressing other equally important factors from the patient perspective, which is central for a truly people-centered approach. As the goal of this article is to present an approach to be replicated and used in operationalizing outcome indicators, we hope it can also be applied to other aspects of quality of care, with potential to improve availability and consistency of patient safety, satisfaction, equity and inclusion data for quality improvement.

The main limitation of the proposed approach is that it has not yet been comprehensively employed in different settings over a long period of time. While the feasibility of selected reference indicators has been confirmed during the country visits, the complete operationalization from identifying constructs, indicators and appropriate measurement method, to collecting data and interpreting analysis of different types of data has not been implemented within one or more humanitarian agencies and responses. The operationalization of this approach for adapting reference indicators and measurement methods to context will be the next phase of this research, including the development of practical guidance on interpretation and data use.

## Conclusion

Monitoring health service coverage, quality and population health status is critical to evaluating effectiveness of humanitarian assistance. While this remains challenging due to multi-faceted constraints characterizing humanitarian settings, it should not be avoided. Adapting measurement methods to each context allows humanitarian actors to gain insight into important health constructs when resources or access limitations may not allow preferred or gold standard approaches. Adopting systematic approaches to choosing alternative indicators and measurement methods can increase the consistency of measures used over time and across locations. By facilitating the performance monitoring of humanitarian action, we can improve our accountability to the affected populations we aim to help and improve the humanitarian response to better meet population health needs. Future humanitarian emergencies will likely become more numerous and complex due to a variety of factors including the climate crisis. Consequently, the need to measure humanitarian action more effectively according to changes in contexts will only become more important.

## Electronic supplementary material

Below is the link to the electronic supplementary material.

Supplementary Material 1

## Data Availability

Not applicable.

## Footnotes

1 Lot Quality Assurance Sampling (LQAS) is a method originally developed for quality control of industrially produced goods by classifying the quality of a given lot as sufficient based on pre-established decision rules. LQAS has been applied to public health to classify the coverage of intervention as acceptable based on a small sample.

## List of abbreviations

ANC: Antenatal care.
BP: Blood Pressure.
DHIS2: District Health Information Software 2.
DRC: Democratic Republic of Congo.
HAUS: Health Access and Utilization survey.
HIS: Health Information System.
HIV: Human Immunodeficiency Virus.
IASC: Interagency Standing Committee.
IAWG: Interagency Working Group.
iRHIS: Integrated Refugee Health Information System.
LQAS: Lot quality assurance sampling.
NGO: Non-governmental organization.
SENS: Standardized Expanded Nutrition Survey.
SARA: Service Availability and readiness assessment.
SMART: Standardized Monitoring and Assessment of Relief and Transitions.
SPA: Service Provision Assessment.
TB: Tuberculosis.
UNHCR: United Nations High Commissioner for Refugees.
UNICEF: United Nations Children’s Fund.
WASH: Water, sanitation and hygiene.
WHO: World Health Organization.
