# Supplementary material for: Measuring results of humanitarian action: adapting public health indicators to different contexts
Source: Confl Health. 2022 Oct 14;16:54. doi: 10.1186/s13031-022-00487-5 (PMC9569100; doi:10.1186/s13031-022-00487-5)
Supplement: Supplementary file 1 — Supplementary Material 1 [file 13031_2022_487_MOESM1_ESM.docx]

Supporting information

Literature review

Table S1: Literature search strategy summary

| **Peer Reviewed Sources** | |
| --- | --- |
| PubMed, EMBASE, SCOPUS  (using MESH terms and EMTREE) | Concept 1: “Humanitarian” or “Warfare and Armed Conflict” or “Relief Work”  AND  Concept 2: “Epidemiologic measurements” or “Epidemiological methods” or “Quality of healthcare” or “Health Indicators” |
| **Grey Literature Sources** | |
| Humanitarian Response Plans | South Sudan  Yemen |
| Coordination bodies | Global Health Cluster  Global Nutrition Cluster  Global WASH Cluster |
| Monitoring frameworks of humanitarian organizations | 5 NGOs (International Committee of the Red Cross, Médecins San Frontières, Action Contre la Faim, Save the Children, International Rescue Committee)  3 UN Agencies (WHO, UNICEF, UNHCR) at HQ and regional levels |
| Monitoring frameworks of donors | 3 donors (UK Department for International Development, US Agency for International Development, World Bank) |
| International guidance and standards | Sphere Standards  Global Health Cluster Core Indicators  WHO 100 Core Health Indicators  Humanitarian Indicators registry  IASC Indicators registry  SMART methodology guidance  Multi-Cluster/Sector Initial Rapid Assessment guidance  IAWG Interagency Field Manual for Reproductive Health in Crises |

Table S2: Framework of public health constructs, sub-constructs and dimensions

| **Construct** | **Sub-constructs** | **Dimensions** | | |
| --- | --- | --- | --- | --- |
|  |  | **Status** | **Coverage** | **Quality** |
| Morbidity | Age specific: newborn, under 5 adolescents | x |  |  |
|  | Cause specific: infectious, chronic, TB, HIV | x |  |  |
| Nutrition | Children under 5 | x |  |  |
|  | Pregnant and lactating women | x |  |  |
| Acute care services | Emergency obstetric care |  | x | x |
|  | Small and sick newborn care |  | x | x |
|  | Sick infant and childcare |  | x | x |
|  | Nutrition (children under 5) |  | x | x |
|  | Gender-based violence |  | x | x |
|  | Emergency medicine/ trauma |  | x | x |
| Chronic care services | Diabetes |  | x | x |
|  | Hypertension |  | x | x |
|  | Tuberculosis |  | x | x |
|  | HIV |  | x | x |
|  | Mental health |  | x | x |
|  | HIV (maternal / newborn) |  | x | x |
| Preventive services | Antenatal care |  | x | x |
|  | Routine labor and delivery, newborn care |  | x | x |
|  | Safe abortion care |  | x | x |
|  | Postnatal care |  | x | x |
|  | Immunization and chemoprevention |  | x | x |
|  | Family planning |  | x | x |
| Preventive practices | Care seeking behaviors |  | x |  |
|  | Preventative practices |  | x |  |
|  | Childcare practices |  | x |  |
| Health System | Human resources |  | x | x |
|  | Health information systems |  | x | x |
|  | Access to essential medicines |  | x | x |
|  | Infection prevention and control |  | x | x |
|  | Service delivery |  | x | x |

Table S3: Reference indicators for each sub-construct/dimension pair by scenario

| **CONSTRUCT** | **SUB-CONSTRUCT** | | **DIMENSION** | **GOLD STANDARD** | **A: ACCESS + /**  **RESOURCES +** | **B: ACCESS - /**  **RESOURCES +** | **C: ACCESS + /**  **RESOURCES -** | **D: ACCESS - /**  **RESOURCES -** |
| --- | --- | --- | --- | --- | --- | --- | --- | --- |
| MORBIDITY | CAUSE SPECIFIC: Infectious with outbreak potential and common IF w high morbidity | | STATUS | Incidence rate | **Surveillance**: number of cases & proportional morbidity |  | **Sentinel sites:** number of cases & proportional morbidity |  |
|  | CAUSE SPECIFIC: Infectious with outbreak potential that reach the alert threshold | | STATUS | Incidence rate | **Surveillance/ EWARN + verification:** Number of new cases | **Surveillance/ EWARN**: Number of new cases | **EWARN + verification:** Number of new cases | **EWARN:** Number of new cases |
|  | CAUSE SPECIFIC: General Chronic | | STATUS | Prevalence of a given chronic disease | **Population based survey**: prevalence of a given chronic disease | **Health facility estimate** of the burden of care: proportion of consultation for chronic diseases. | **Rapid population-based survey**: prevalence of a given chronic disease | **Sentinel sites (Health facilities)**: proportion of consultation for chronic diseases. |
|  | CAUSE SPECIFIC:  TB | | STATUS |  | **Passive screening (HIS):** Number of new cases |  | **Sentinel sites**: number of new cases |  |
|  | CAUSE SPECIFIC: HIV | | STATUS |  | **Population based survey:** prevalence of HIV | **HIS**: Prevalence among ANC women | **ANC surveillance**: Prevalence among ANC women | **Sentinel ANC sites**: number of patients tested positive in the reporting period |
|  | AGE SPECIFIC: Newborn | | STATUS | Prevalence of low birth weight among neonates: Number of live-born neonates with weight less than 2500 g obtained within 24 hours of birth, regardless of gestational age / total number of live births. | **Health facility based (HIS)**: proportion of live births with weight less than 2500 gm at birth |  | **Sentinel sites**: proportion of live births with weight less than 2500 gm at birth |  |
|  | AGE SPECIFIC: Adolescents | | STATUS | **Adolescent birth rate:** number of births to women aged 15 to 19 per 1000 women in that age group per year. | **Population based birth rate:** number of births to women aged 15 to 19 per 1000 per unit of time | **Facility estimate**s: proportion of births to women aged 15 to 19 over the total facility births | **Administrative population estimate**: number of births to women aged 15 to 19 per 1000 per unit of time | **Sentinel sites of health facilities:** proportion of births to women aged 15 to 19 over the total facility births |
| NUTRITIONAL STATUS | PREGNANT LACTATING WOMEN | | STATUS | Prevalence of undernutrition in pregnant and non-pregnant women. | **Population based survey:** Proportion of women in reproductive age with MUAC< 23 cm or national threshold | **HIS or** **Health facility surveys**: Proportion of women going to ANC visits with MUAC< 23 cm or national threshold | **Rapid population based (LQAS)**: Number of women in reproductive age with MUAC< 23 cm or national threshold exceeds or meets decision threshold | **Sentinel site of health facilities:** Proportion of women going to ANC visits with MUAC< 23 cm or national threshold |
|  | UNDER FIVE YEARS | | STATUS | Proportion of children with WHZ < -2 SD or MUAC <125mm (GAM), o BP oedema; or WHZ < -3 or MUAC<115 mm, or BP oedema (SAM) (population based survey) | **Population based survey**: Prevalence of GAM and SAM: Proportion of children with WHZ < -2 SD or MUAC <125mm (GAM), o BP oedema; or WHZ < -3 or MUAC<115 mm, or BP oedema (SAM) | **Nutrition program data + administrative population estimate**: Number of children admitted to therapeutic nutrition program / administrative estimate of under 5 population | **Rapid population-based survey**: Proportion of children with WHZ < -2 SD or MUAC <125mm (GAM), o BP oedema; or WHZ < -3 or MUAC<115 mm, or BP oedema (SAM) | **Sentinel sites (Nutrition programs):** Admissions to nutrition programs / estimated under 5 population |
| ACUTE CARE SERVICES | EMERGENCY OBSTETRIC CARE (EmOC) | | COVERAGE | Population based. Proportion of women with major direct obstetric complications that receive care (met need for EmOC) in a given period | ACCESS TO POPULATION: **Population based survey + health facility assessment**: Proportion of deliveries that occurred in a health facility that demonstrates capacity to provide BEmOC/ CEmOC signal functions in a given period | ACCESS TO HEALTH FACILITIES: **HIS + HF assessment + administrative population estimate**: Proportion of facility births that occurred in a health facility that demonstrates capacity to provide BEmOC/ CEmOC signal functions over the estimated number of live births in population | ACCESS to HEALTH FACILITIES: **HF estimate + HF assessment**: Proportion of facility births that occurred in a health facility that demonstrates capacity to provide BEmOC/ CEmOC signal functions in a given period | **Sentinel sites of health facilities:** Proportion of facilities that report providing all BEmOC/ CEmOC signal functions in the past 3 months |
|  |  |  | QUALITY | Proportion of women with severe pre-eclampsia or eclampsia that promptly receive appropriate interventions, according to WHO guidelines. | **Random sample of facilities (in person):** Percent of women with severe pre-eclampsia /eclampsia that received a loading dose of magnesium sulfate in a given period (in person review of records) | **HIS (all reporting health facilities) or Random sample of facilities (remote)**: Percent of facilities with recommended anticonvulsant (magnesium sulfate) in stock | **LQAS sample of facilities:** Yes/No: Number of facilities with recommended anticonvulsant in stock meets or exceeds LQAS threshold. | **Sentinel sites of health facilities:** Percent of facilities with recommended anticonvulsant in stock |
|  | SMALL AND SICK NEWBORN CARE | | COVERAGE | Facility based.  Proportion of small, sick, or preterm newborns that received care (resuscitation, KMC, treatment of PSCBI, NICU) in a given period (met need for care to address small and sick newborns) | ACCESS TO POPULATION: **Population based survey + health facility assessment**: Proportion of births that occurred in a health facility that demonstrates capacity to provide care sick newborns in a given period | ACCESS TO HEALTH FACILITIES: **HIS + HF assessment + administrative population estimate**: Proportion of facility births that occurred in a health facility that demonstrates capacity to provide care for sick newborns in a given period | ACCESS to HEALTH FACILITIES: **HF estimates + HF assessment**: Proportion of births that occurred in a health facility that demonstrates capacity to provide care for sick newborns in a given period | **Sentinel sites of health facilities**: Proportion of facilities that report capacity to provide neonatal resuscitation |
|  |  |  | QUALITY | Proportion of newborns diagnosed with PSBI who received injectable antibiotics, according to WHO guidelines | **Random sample of facilities (in person)**: Percent of newborns diagnosed with PSBI who received at least one dose of antibiotics at the facility | **HIS (all reporting health facilities) or random sample of health facilities (remote):** Percent of facilities with recommended antibiotics in stock | **LQAS sample of health facilities: Yes/No**: Number of newborns with PSBI who received at least one dose of antibiotics at the facility meets or exceeds LQAS threshold. | **Sentinel sites of health facilities**: Percent of facilities with recommended antibiotics in stock |
|  | GENDER-BASED VIOLENCE | | COVERAGE |  | **Random sample of health facilities (in person)**: Percent of rape survivors who report to health facilities/ workers within 72 hours and receive HIV post-exposure prophylaxis (PEP) within 72 hours of an incident occurring, over the total number of reported rape cases | **HIS (all reporting health facilities) or random sample of health facilities (remote)**: Percent of rape survivors who report to health facilities/ workers within 72 hours and receive HIV post-exposure prophylaxis (PEP) within 72 hours of an incident occurring, over the total number of reported rape cases | **Rapid sample of health facilities (LQAS in person):** Number of rape survivors who report to health facilities/ workers within 72 hours and receive HIV post-exposure prophylaxis (PEP) within 72 hours of an incident occurring meets or exceeds LQAS threshold | **Sentinel sites (health facilities):** Proportion of health facilities that report providing PEP services for rape survivor in the past three months |
|  |  |  | QUALITY |  | **Random sample of health facilities (in person)**: Percent of rape survivors who report to health facilities/ workers within 72 hours and receive appropriate medical care (PEP within 72 hours, EC 120 Hours, STI 2 weeks) over the number of rape survivors who reported within 72 hours | **HIS (all reporting health facilities) or random sample of health facilities (remote**): Percent of rape survivors who report to health facilities/ workers within 72 hours and receive HIV post-exposure prophylaxis (PEP) within 72 hours of an incident occurring (over the number of rape survivors who reported within 72 hours) | **LQAS sample of health facilities**: Yes/No: Number of rape survivors in the emergency area who report to health facilities/workers within 72 hours and receive appropriate medical care (PEP within 72 hours, EC 120 Hours, STI 2 weeks) meets or exceeds LQAS threshold. | **Sentinel sites of health facilities:** Percent of rape survivors in the emergency area who report to health facilities/ workers within 72 hours and receive appropriate medical care (PEP within 72 hours, EC 120 Hours, STI 2 weeks) (over the number of rape survivors who reported within 72 hours) |
|  | NUTRITION (UNDER 5) | | COVERAGE | Proportion of SAM children undergoing treatment + recovering children over the total number of SAM children + recovering children in the community | **Population based estimate:** Period coverage of treatment programs (SQUEAC) | **Administrative coverage (Program data + expected SAM prevalence)**: proportion of SAM children enrolled into a treatment program | **Rapid population based**: Number of service delivery units with adequate level of coverage meets or exceeds LQAS threshold (SLEAC) | **Availability coverage**: Proportion of health facilities providing CMAM programs |
|  |  |  | QUALITY | CMAM indicators | **Random sample of health facilities** (in person): Proportion of charts reviewed where child received correct amount of RUTF per national guidelines | **HIS:** comparison between RUTF utilization and SAM caseload | **LQAS sample of health facilities**: Yes/No: Number of health facilities where children received the correct amount of RUTF per national guidelines meets or exceeds LQAS threshold | **Sentinel sites:** proportion of days in the reporting period with a stock out of RUTF |
|  | SICK INFANT AND CHILD CARE | |  | Malaria endemic |  |  |  |  |
|  |  |  | COVERAGE | Proportion of children under five with fever who were tested for malaria | ACCES TO POPULATION: **Population based survey**: proportion of children under 5 with fever who were tested for malaria (who had a finger or heel stick) | ACCESS + TO HEALTH FACILITIES and RESOURCE +: **HIS or Random sample of health facilities (remote)**: Proportion of under 5 confirmed malaria cases who received antimalarial treatment after diagnosis (clinical and lab) | ACCESS + and LIMITED RESOURCE: LQAS population based survey: Yes/No: Number of children under five with fever who were tested for malaria (who had a finger or heel stick) meets or exceeds LQAS threshold. | Sentinel sites (health facilities) (remotely): Proportion of under five malaria cases who received antimalarial treatment after diagnosis (clinical and lab) |
|  |  |  |  | Malaria NON endemic |  |  |  |  |
|  |  |  | COVERAGE | Percent of children under five with cough and difficulty breathing who sought care from an appropriate provider | **Population-based survey**: Percent of children under five with cough and difficulty breathing who sought care from an appropriate provider | **HIS or Random sample of health facilities (remote)**: Proportion of under five year old clinically or laboratory confirmed pneumonia cases who received antibiotic treatment after diagnosis | LQAS population based survey: Yes/No: Number of children under five with cough and difficulty breathing who sought care from an appropriate provider meets or exceeds LQAS threshold. | **Sentinel sites (health facilities) (remotely):** Percent of under five year old clinically or laboratory confirmed pneumonia cases who received antibiotic treatment after diagnosis |
|  |  |  |  | Malaria endemic |  |  |  |  |
|  |  |  | QUALITY |  | **Random sample of facilities (in person**): Proportion of facilities managing malaria cases under 5 according to national protocol/clinical guidance (or IMCI guidelines if national protocols are not available) | **Random sample of facilities (remote)**: Proportion of facilities managing malaria cases under 5 according to national protocol/clinical guidance (or IMCI guidelines if national protocols are not available) | **LQAS sample of facilities Yes/No:** Number of facilities managing malaria cases under 5 according to national protocol/clinical guidance (or IMCI guidelines if national protocols are not available) meets or exceeds LQAS threshold | **Sentinel sites** (remote): Proportion of facilities managing malaria cases under 5 according to national protocol/clinical guidance (or IMCI guidelines if national protocols are not available) |
|  |  |  |  | Malaria NON endemic |  |  |  |  |
|  |  |  | QUALITY | Proportion of sick children managed according to IMCI guidelines. | **Random sample of facilities (in person)**: Proportion of facilities managing pneumonia cases under 5 according to national protocol/clinical guidance (or IMCI guidelines if national protocols are not available) | **Random sample of facilities (remote)**: Proportion of facilities managing pneumonia cases under 5 according to national protocol/clinical guidance (or IMCI guidelines if national protocols are not available) | **LQAS sample of facilities: Yes/No:**: Number of facilities managing pneumonia cases under 5 according to national protocol/clinical guidance (or IMCI guidelines if national protocols are not available) meets or exceeds LQAS threshold | **Sentinel sites (remote)**: Proportion of facilities managing pneumonia cases under 5 according to national protocol/clinical guidance (or IMCI guidelines if national protocols are not available) |
|  | TRAUMA/ EMERGENCY SURGERY/ MEDICINE | | COVERAGE |  | **Census of health facilities expected to provide the service (in person)**: Proportion of health facilities that demonstrate capacity to provide Bellwether procedures (caesarian delivery, laparotomy and treatment of open fracture) in the last one month | **HIS (all reporting facilities expected to provide the service)**: Proportion of health facilities that reported providing Bellwether procedures (caesarian delivery, laparotomy and treatment of open fracture) in the last one month | **Census of health facilities expected to provide this service**: Proportion of health facilities that demonstrate capacity to provide Bellwether procedures (caesarian delivery, laparotomy and treatment of open fracture) in the last one month | **Sentinel sites of health facilities expected to provide the service:** Proportion of health facilities that reported providing Bellwether procedures (caesarian delivery, laparotomy and treatment of open fracture) in the last one month |
|  |  |  | QUALITY |  | **Census of health facilities expected to provide the service: (in person):** Proportion of facilities able to provide routine intra operative monitoring & have trained anesthesiologist & conduct routine sterilization of the operative venue and equipment. | **Census or random sample of facilities expected to provide the service (remote)**: Proportion of facilities that have at least one trained anesthesiologist | **Census or random sample of facilities expected to provide the service (in person):** Proportion of facilities able to provide routine intra operative monitoring & have trained anesthesiologist & conduct routine sterilization of the operative venue and equipment. | **Sentinel sites of health facilities expected to provide the service:** Proportion of facilities that have at least one trained anesthesiologist |
| CHRONIC CARE SERVICES | HYPERTENSION | | COVERAGE |  | ACCES TO POPULATION: **Population based survey**: Percentage of adults with hypertension who received treatment in the last 12 months | ACCESS TO HEALTH FACILITIES: **HIS or Random sample of health facilities (in person)**: Proportion of adult with hypertension under treatment | ACCES TO POPULATION: **LQAS Population based survey**: Yes/No: Number of adults with hypertension who have received treatment in the last 12 months meets or exceeds LQAS threshold | **Sentinel sites**: Proportion of health facilities with blood pressure cuff/machines and no stockout of anti-hypertension medications in the last 6 months |
|  |  |  | QUALITY |  | **Random sample of health facilities (in person):** Proportion of adult hypertension patients who receive appropriate treatment according to protocol | **HIS or Random sample of health facilities (remote)**: Proportion of health facilities with blood pressure cuff/machines and no stockout of anti-hypertension medications in the last month | **LQAS sample of health facilities**: Yes/No: Number of health facilities managing hypertension patients according to protocol meets or exceeds LQAS threshold | **Sentinel facilities:** Proportion of clinics with blood pressure cuff/machines and no stockout of anti-hypertension medications in the last month |
|  | DIABETES | | COVERAGE |  | ACCES TO POPULATION: **Population based survey**: Percentage of adults with diabetes who received/ are under treatment in the last 12 months | ACCESS TO HEALTH FACILITIES: **HIS or Random sample of health facilities (in person)**: Proportion of adults with diabetes who are under treatment | ACCES TO POPULATION: **LQAS Population based survey**: Yes/No: Number of adults with diabetes who received/ are under treatment in the last 12 months meets or exceeds LQAS threshold | **Sentinel sites**: Proportion of health facilities with glucometers and no stockout of oral anti diabetes medicaments |
|  |  |  | QUALITY |  | **Random sample of health facilities (in person):** Proportion of patients who receive appropriate treatment according to protocol | **HIS or random sample of health facilities (remote)**: Proportion of health facilities with glucometers and no stockout of oral anti diabetes medication in the last month | **LQAS sample of health facilities**: Number of health facilities in which the quality of diabetes care is sufficient meets or exceed LQAS threshold | **Sentinel facilities:** Proportion of health facilities with glucometers and no stockouts of oral anti diabetes medication in the last month |
|  | NCD treated as CD - HIV | | COVERAGE |  | *See separate table at the bottom of the table (6 options instead of 4 are provided)* | | | |
|  |  |  | QUALITY |  | **Random sample of HF (in person)**: Proportion of patients who receive appropriate treatment according to protocol | **HIS or Random sample of HF (remote)**: Proportion of health facilities with HIV tests and with no stock out of ART according to national reporting frequency | **LQAS sample of health facilities**: Yes/No: Number of patients on HIV care who receive appropriate treatment according to protocol meets or exceeds LQAS threshold | **Sentinel site:** Proportion of health facilities with HIV tests and with no stock out of ART in the last month |
|  | NCD treated as CD - TB | | COVERAGE |  | **HIS (all reporting facilities):** Proportion of confirmed TB cases (clinically or laboratory confirmed) who have initiated treatment |  | **Sentinel sites:** Proportion of confirmed TB cases (clinically or laboratory confirmed) who have initiated treatment |  |
|  |  |  | QUALITY |  | **Random sample of HF (in person):** Proportion of the cohort of patients who should have finished treatment in the month prior to the assessment | **Random sample of HF (remote):** Proportion of the cohort of patients who should have finished treatment in the month prior to the assessment (retrospective assessment of the cohort) | **LQAS sample of health facilities: Yes/No:** Number of the cohort of patients who should have finished treatment in the month prior to the assessment meets or exceeds LQAS threshold | **Sentinel sites:** Proportion of the cohort of patients who should have finished treatment in the month prior to the assessment (retrospective assessment of the cohort) |
|  | HIV (Maternal/ Newborn) | | COVERAGE | Proportion of pregnant women living with HIV who received most efficacious antiretroviral to reduce the risk of mother-to-child transmission of HIV. | **Random sample of HF (in person)**: Proportion of pregnant women with known HIV status at ANC (includes those who already knew their HIV status prior to ANC and those who were tested and received results at ANC1) | **HIS from all health facilities**: Proportion of pregnant women with known HIV status at ANC (includes those who already knew their HIV status prior to ANC and those who were tested and received results at ANC1) | **LQAS sample of health facilities**: Yes/No: Number of pregnant women with known HIV status at ANC (includes those who already knew their HIV status prior to ANC and those who were tested and received results at ANC1) meets or exceeds LQAS threshold | **Sentinel sites (health facilities)**: Proportion of pregnant women with known HIV status at ANC (includes those who already knew their HIV status prior to ANC and those who were tested and received their results at ANC1) |
|  |  |  | QUALITY |  | **Random sample of HF (in person):** Proportion of HIV-positive pregnant women who received ART to reduce the risk of mother-to-child-transmission during pregnancy | **HIS from all health facilities**: Proportion of HIV-positive pregnant women who received ART to reduce the risk of mother-to-child-transmission during pregnancy | **LQAS sample of health facilities: yes/No:** Number of HIV-positive pregnant women who received ART to reduce the risk of mother-to-child-transmission during pregnancy meets or exceeds LQAS threshold | **Sentinel sites (health facilities) (remotely):** Proportion of HIV-positive pregnant women who received ART to reduce the risk of mother-to-child-transmission during pregnancy |
|  | MENTAL HEALTH | | COVERAGE |  | **Survey of service providers (in person):** Proportion of medical facilities, social services facilities and community programs that offer focused MHPSS programs (such as MHGap) | **Program estimate:** Proportion of medical facilities, social services facilities and community programs that offer focused MHPSS programs (such as MHGap) | **LQAS survey of service providers: Yes/No:** Number of medical facilities, social services facilities and community programs that offer focused MHPSS programs (such as MHGap) meets or exceeds LQAS threshold | **Sentinel sites of service providers**: Proportion of medical facilities, social services facilities and community programs that offer focused MHPSS programs (such as MHGap) |
|  |  |  | QUALITY |  | **Random sample of providers /health facilities (in person):** Percentages of medical facilities, social services facilities and community programs who have staff trained in the last two years to identify mental disorders and to support people with mental health and psychosocial problems | **Random sample of service providers / health facilities (remote)**: Percentages of medical facilities, social services facilities and community programs who have staff trained in the last two years to identify mental disorders and to support people with mental health and psychosocial problems | **LQAS sample of service providers / health facilities**: Yes/No: Number of medical facilities, social services facilities and community programs who have staff trained in the last two years to identify mental disorders and to support people with mental health and psychosocial problems meets or exceeds the LQAS threshold | **Sentinel site**: Percentages of medical facilities, social services facilities and community programs who have staff trained in the last two years to identify mental disorders and to support people with mental health and psychosocial problems |
| PREVENTIVE SERVICES | ANTENATAL CARE | | COVERAGE | Population based survey: percent of women with a live birth within a given period with at least four antenatal care visits with skilled health personnel during pregnancy | **Population based estimate**: Proportion of women who reported attending antenatal care with skilled health personnel at least four times during most recent pregnancy | **HIS**: Percent of women who attended antenatal care with skilled health personnel at least four times during pregnancy in the reporting period | **LQAS population based: Yes/No:** Number of women who reported attending antenatal care with skilled health personnel at least four times during pregnancy meets or exceeds LQAS threshold. | **Sentinel sites (health facilities):** Proportion of women who attended antenatal care with skilled health personnel at least four times during pregnancy in the reporting period |
|  |  |  | QUALITY | Facility based. Proportion of women screened for high blood pressure during first antenatal care visit in a given period. | **Random sample of health facilities (in person)**: Percent of ANC clients screened for high blood pressure | **Random sample of health facilities (remote)**: Percent of ANC clients screened for high blood pressure | **LQAS sample of facilities: Yes/No:** Number of facilities where ANC clients are screened for high blood pressure meets or exceeds LQAS threshold | **Sentinel sites (remote):** Percent of facilities contacted that have on-site functioning device for high blood pressure screening |
|  | FAMILY PLANNING | | COVERAGE | Contraceptive Prevalence Rate: The percent of women of reproductive age (15-49) who are using (or whose partner is using) a contraceptive method at a particular point in time | **Population based survey**: Proportion of the demand for family planning satisfied with modern methods | **HIS or survey of health facilities (remote):** % of new FP clients at facilities who adopt oral contraceptive pills, injectables, or long acting reversible methods (implants, IUD) or permanent methods (TL), out of the total new FP clients | **LQAS population based: Yes/No:** Number of non-pregnant married/ in a union women of reproductive age (15-49) and who are using (or whose partner is using) a modern method of contraception meets or exceeds LQAS threshold. | **Sentinel sites (health facilities):** Proportion of facilities providing oral contraceptive pills, injectable or at least one long acting reversible method |
|  |  |  | QUALITY | HH Survey: Percent of women of reproductive age (WRA) (15-49) currently using a modern contraceptive method who report that they obtained their contraceptive method of choice, at a particular point in time | **Random sample of health facilities (in person)**: Percent of service delivery points offering a mixture of short-acting modern contraceptive and long-acting reversible contraceptive methods at the time of assessment | **HIS or random sample of health facilities (remote)**: Percent of service delivery points offering a mixture of short-acting modern contraceptive and long-acting reversible contraceptive methods at the time of assessment | **LQAS sample of health facilities: Yes/No:** Number of service delivery points offering a mixture of short-acting modern contraceptive and long-acting reversible contraceptive methods meets or exceeds LQAS threshold | **Sentinel facilities:** Number of facilities (out of total) offering a mixture of short-acting modern contraceptive and long-acting reversible contraceptive methods |
|  | IMMUNIZATION AND CHEMOPREVENTION | | COVERAGE | Population-based estimate of the proportion of children 6 to 59 months (or 6 months to 15 years) who have received measles vaccination | **Population-based** **survey**: proportion of children 6 to 59 months (or 15 years) who have received measles vaccination | **HIS + Administrative estimate**: Proportion of children less 12 months who have received measles vaccination | **LQAS population based: es/No:** Number of eligible children 6 to 59 months (or to 15 years) who have received measles vaccination per site meets or exceeds LQAS threshold. | **Sentinel sites (health facilities):** Proportion of children less than 12 months who have received measles vaccination |
|  |  |  | QUALITY |  | **Random sample of facilities (in person):** Proportion of facilities with functioning cold chain (refrigerator at correct temperature + temp chart, or viable vaccine vial monitors) | **HIS:** Proportion of reporting facilities with functioning cold chain (refrigerator at correct temperature + temp chart, or viable vaccine vial monitors) | **LQAS sample of health facilities: Yes/No:** Number of facilities with functioning cold chain (refrigerator at correct temperature + temp chart, or viable vaccine vial monitors) meets or exceeds LQAS threshold | **Sentinel sites:** Percent of facilities with functioning cold chain (refrigerator at correct temperature + temp chart, or viable vaccine vial monitors) |
|  | ROUTINE CARE DURING LABOR AND CHILDBIRTH, NEWBORN CARE (DAY OF BIRTH) | | COVERAGE | Proportion of births attended by skilled health personnel (SDG Indicator 3.1.2) | **Population-based survey**: proportion of births in a health facility in a given time period / area | **HIS + Administrative estimate**: proportion of births in a health facility in a given time period / area | **LQAS population based survey**: Yes/No: Number of women who gave birth at a health facility in a given time period / area meets or exceeds LQAS threshold. | **Sentinel sites (HF)**: Number of live births in each contacted health facility in the reporting period |
|  |  |  | QUALITY | Facility based: Percent of women/girls giving birth at a facility who receive a uterotonic immediately after birth for prevention of postpartum hemorrhage | **Random sample of health facilities (in person)**: Percent of women/girls giving birth at a facility who receive a uterotonic immediately after birth for prevention of postpartum hemorrhage | **Random sample of facilities (remotely)**: Percent of women/girls giving birth at a facility who receive a uterotonic immediately after birth for prevention of postpartum hemorrhage | **LQAS sample of health facilities: Yes/No:** number of facilities where women/girls giving birth received a uterotonic immediately after birth for prevention of postpartum hemorrhage (observation or record review) meets or exceeds LQAS threshold | **Sentinel sites:** Percent of facilities with uterotonic in stock for average number of deliveries on day of assessment |
|  | SAFE ABORTION CARE | | COVERAGE | *Facility based.* Proportion of service delivery points that demonstrate capacity to provide safe termination of pregnancy services, to the full extent of the law, in a given period | **Random sample of health facilities (in person):** Percent of health facilities that provide basic SAC signal functions in a given period | **Random sample of health facilities (remotely)**: Percent of health facilities that report provision of basic SAC signal functions in a given period | **LQAS sample of health facilities: Yes/No:** Number of facilities that provide basic SAC signal functions in a given period meets or exceeds LQAS threshold | **Sentinel sites (health facilities) (remotely**): Percent of health facilities that report provision of basic SAC signal functions in a given period |
|  |  |  | QUALITY | *Facility based.* Proportion of women with spontaneous or induced abortion who receive appropriate interventions, according to WHO guidelines | **Census of facilities providing PAC:** Proportion of PAC clients who received counseling on post-pregnancy contraception in a given period. | **Random sample of health facilities (remote)**: Percent of PAC clients who received counseling on post-pregnancy contraception in a given period. | **Census of facilities providing PAC + LQAS:**  Number of PAC clients who received counseling on post-pregnancy contraception meets or exceeds LQAS threshold in a given period. | **Sentinel facilities providing PAC:** Proportion of facilities providing PAC that document the number of post-abortion clients receiving contraception services or counseling in a given period. |
|  | POSTNATAL CARE (PNC) | | COVERAGE | Population-based estimate of the percent of mothers and newborns attended by a health care provider during the first 48 hours following birth (but after the immediate postpartum check) in a given time period/ area | **Population-based survey:** proportion of mothers and newborns attended by a health care provider during the first 48 hours following birth in a given time period | **HIS + Administrative estimate**: Proportion of mothers and newborns attended by a health care provider during the first 48 hours following birth in the reporting period | **LQAS population based**: Yes/No: Number of mother / newborn pairs attended by a health care provider during the first 48 hours following birth in a given time period meets or exceeds LQAS threshold. | **Sentinel sites (health facilities)**: Proportion of health care facilities that provided PNC service in the month prior to the assessment out of those contacted |
|  |  |  | QUALITY | Population-based percentage of women who report they received family planning counselling during the 1st PNC visit for mom and/or baby | **Population-based survey**: percentage of women who received PNC reporting they received breastfeeding counselling by skilled personnel during the PNC visits for mom and/or baby | **HIS or Random sample of health facilities (remote)**: percentage of women who report they received breastfeeding counselling by skilled personnel during the PNC visits for mom and/or baby | **LQAS population based survey:** Number of women, among those tho received PNC, who report they received breastfeeding counselling by skilled personnel during the PNC visit for mom and/or baby meets or exceeds LQAS threshold | **Sentinel site (health facilities):** Proportion of facilities that report providing BF counselling during PNC |
| PREVENTIVE PRACTICES | CARE SEEKING BEHAVIOUR | | COVERAGE | Proportion of children under the age of 5 with suspected pneumonia taken to an appropriate health facility or provider | **Population based survey:** Proportion of children under the age of 5 with suspected pneumonia/ symptoms of ARI taken to a health facility or provider | **HIS or random sample of health facilities (in person)**: Number of outpatient and inpatient visits for suspected pneumonia | **LQAS population based**: Yes/No: Number of children under the age of 5 with suspected pneumonia/ symptoms of ARI (or fever if a malaria endemic region) taken to an appropriate health facility or provider meets or exceeds LQAS threshold | **Sentinel sites (health facilities)**: Number of outpatient and inpatient visits with a diagnosis of pneumonia |
|  | PREVENTIVE PRACTICES | | COVERAGE | Proportion of men, women, boys and girls who wash hands with water and soap or substitute after contact with feces and before contact with food and water (observation within a population-based survey) | **Population based survey:** Proportion of households with fixed handwashing facility on the premises with water and soap which show evidence of use & regular maintenance | **Random sample of health facilities (in person)**: proportion of women exiting the health facility who report having received / having soap at home | **LQAS population based: Yes/No:** Number of households with a fixed handwashing facility with water and soap which show evidence of use and regular maintenance meets or exceeds LQAS threshold | **Program estimate:** proportion of targeted households who received soap |
|  | CHILD CARE PRACTICES | | COVERAGE | Proportion of children born in the last 24 months who were put to breast within one hour from birth. | **Population based survey:** Proportion of children born in the last 12 months who were put to breast within one hour/ soon after from birth | **HIS or Random sample of health facilities (in person)**: Proportion of children born in the last 12 months who were put to breast within one hour from/ soon after birth | **LQAS population based survey: Yes/No**: Number of children born in the last 12 months who were put to breast within one hour from/ soon after birth meets or exceeds LQAS threshold | **Sentinel sites of health facilities**: number of facilities contacted who have a breastfeeding support policy in place out of the facilities contacted. Alternative: number of facilities contacted who have at least one health care staff who received a training on essential newborn care out of the facilities contacted |
| HEALTH SYSTEM | HUMAN RESOURCES | | COV & QUAL |  | **Random sample of health facilities (in person)**: Number of outpatient consultations per clinician per day per administrative unit | **HIS:** Number of outpatient consultations per clinician per day per administrative unit | **LQAS sample of Health facilities**: yes/No: Number of outpatient consultations per clinician per day per administrative unit meets or exceed LQAS threshold | **Sentinel sites (health facilities):** Number of consultations per clinician per day per administrative unit |
|  | SERVICE DELIVERY | | COV & QUAL |  | **Random sample of Health facilities (in person)**: proportion of health facilities that meet all tracer criteria for basic amenities | **Random sample of HF (remote)**: proportion of health facilities that meet all tracer criteria for basic amenities | **LQAS sample of health facilities**: yes/No: Number of health facilities that report meeting all tracer criteria for basic amenities meets or exceeds LQAS threshold | **Sentinel sites:** proportion of health facilities that report meeting all tracer criteria for basic amenities |
|  | HEALTH INFORMATION SYSTEM | | COVERAGE |  | **All health facilities included in the HIS:** Proportion of health facilities that reported in the last reporting period | | | |
|  |  |  | QUALITY |  | **Random sample of health facilities (in person):** Proportion of HF for which the patient caseload matches the one reported into HIS for measles vaccination in the last reporting period | **Random sample of health facilities (remote)**: proportion of health facilities with data quality assessment carried out in the last year using agreed quality criteria such as WHO's Data Quality Review toolkit | **LQAS sample of health facilities: Yes/No:** Number of health facilities for which the number of patient caseload matches the one reported into HIS for measles vaccinations in the last reporting period meets or exceeds LQAS threshold | **Sentinel sites (health facilities):** proportion of health facilities reporting having conducted data quality assessments carried out in the last year using agreed quality criteria such as WHO's Data Quality Review toolkit |
|  | INFECTION PREVENTION AND CONTROL | | COV&QUAL |  | **Random sample of health facilities (in person):** Percent of health facilities where all items needed to implement standard precautions for infection prevention are available | **Random sample of health facilities (remote):** Percent of health facilities where all items needed to implement standard precautions for infection prevention are available | **LQAS sample of health facilities: Yes/No:** Number of health facilities where all items needed to implement standard precautions for infection prevention are available meets or exceeds LQAS threshold | **Sentinel sites (health facilities) (remotely):** Percent of health facilities where all items needed to implement standard precautions for infection prevention are available |
|  | ESSENTIAL MEDICINES | | COV&QUAL |  | **Random sample of health facilities (in person):** Proportion of health facilities that did not report any stock out of WHO-recommended core list of medicines over the last month | **Logistic or HIS or random sample of health facilities (remote)**: Proportion of health facilities that did not report any stock out of WHO-recommended core list of medicines over the last month | **LQAS sample of health facilities**: Number of health facilities that did not report any stock out of WHO-recommended core list of medicines over the last month meets or exceeds LQAS threshold | **Sentinel sites**: Proportion of health facilities that did not report any stock out of WHO-recommended core list of medicines over the last month |
|  |  |  |  |  |  |  |  |  |
|  | Continued table S3: Coverage of HIV | | | |  |  |  |  |
|  | NCD treated as CD – HIV | | COVERAGE |  | **Functioning HIS** | **Facility providing ARV** |  |  |
|  |  |  |  | **Resources ++**  **(time and funding/HR)** | **A: HIS + population based:** Proportion of HIV positive patients on treatment | **D: HF + population based:** Proportion of HIV positive patients on treatment |  |  |
|  |  |  |  |  | Denominator: number of persons estimated to be HIV positive in the area of interest (based on recent population-based survey) | |  |  |
|  |  |  |  | **Resources +**  **(little time or funding/ HR)** | **B: HIS + ANC sentinel site:** Proportion of HIV positive patients on treatment | **E: HF + ANC sentinel site:** Proportion of HIV positive patients on treatment |  |  |
|  |  |  |  |  | Denominator**:** number of persons estimated to be HIV positive in the area of interest (based on ANC sentinel surveillance estimates) | |  |  |
|  |  |  |  | **Resources –**  **(little time or funding/ HR)** | **C: HIS + ANC facility estimates:** Proportion of HIV positive patients on treatment | **F: HF + ANC facility estimate:** Proportion of HIV positive patients on treatment |  |  |
|  |  |  |  |  | Denominator: number of persons estimated to be HIV positive based on ANC facility estimate | |  |  |
